# Are AMI Patients with Comorbid Mental Illness More Likely to be Admitted to Hospitals with Lower Quality of AMI Care?

**DOI:** 10.1371/journal.pone.0060258

**Published:** 2013-04-02

**Authors:** Xueya Cai, Yue Li

**Affiliations:** 1 Department of Biostatistics and Computational Biology, University of Rochester School of Medicine and Dentistry, Rochester, New York, United States of America; 2 Department of Public Health Sciences, University of Rochester School of Medicine and Dentistry, Rochester, New York, United States of America; Sapienza University of Rome, Italy

## Abstract

**Objective:**

Older patients with comorbid mental illness are shown to receive less appropriate care for their medical conditions. This study analyzed Medicare patients hospitalized for acute myocardial infarction (AMI) and determined whether those with comorbid mental illness were more likely to present to hospitals with lower quality of AMI care.

**Methods:**

Retrospective analyses of Medicare claims in 2008. Hospital quality was measured using the five “Hospital Compare” process indicators (aspirin at admission/discharge, beta-blocker at admission/discharge, and angiotension-converting enzyme inhibitor or angiotension receptor blocker for left ventricular dysfunction). Multinomial logit model determined the association of mental illness with admission to low-quality hospitals (rank of the composite process score <10^th^ percentile) or high-quality hospitals (rank>90^th^ percentile), compared to admissions to other hospitals with medium quality. Multivariate analyses further determined the effects of hospital type and mental diagnosis on outcomes.

**Results:**

Among all AMI admissions to 2,845 hospitals, 41,044 out of 287,881 patients were diagnosed with mental illness. Mental illness predicted a higher likelihood of admission to low-quality hospitals (unadjusted rate 2.9% vs. 2.0%; adjusted odds ratio [OR]1.25, 95% confidence interval [CI] 1.17–1.34, p<0.01), and an equal likelihood to high-quality hospitals (unadjusted rate 9.8% vs. 10.3%; adjusted OR 0.97, 95% CI 0.93–1.01, p = 0.11). Both lower hospital quality and mental diagnosis predicted higher rates of 30-day readmission, 30-day mortality, and 1-year mortality.

**Conclusions:**

Among Medicare myocardial infarction patients, comorbid mental illness was associated with an increased risk for admission to lower-quality hospitals. Both lower hospital quality and mental illness predicted worse post-AMI outcomes.

## Introduction

Acute myocardial infarction (AMI) is one of the most severe forms of heart disease and contributes significantly to the morbidity and mortality in the United States. In 2006, almost one million Americans were diagnosed with new or recurrent myocardial infarction and over 140 thousand died of AMI [1]. Persons with mental illness are at increased risks for developing heart disease, and have worse prognosis following a cardiac event [2], [3], [4], [5]. For example, depression may lead to a 70 percent increased lifetime risk for fatal or non-fatal AMI [3].

A growing body of literature has also suggested that compared to the general population, mentally ill patients tend to receive less optimal physician and hospital care for common medical conditions [6], [7], [8]. In particular, after the onset of AMI, patients with comorbid mental illness are less likely to receive medications with known benefit of reducing morbidity and mortality (e.g., beta-blockers at hospital admission for AMI) [9], [10], [11], [12]. However, beyond the documentation of reduced quality of medical care among the mentally ill, relatively less is known about whether this phenomenon is a function of patient factors (e.g., cognitive impairments), provider factors (e.g., quality of hospital care), or both.

Several studies reported that mentally-ill patients were less compliant with prescribed treatments for their medical conditions, which may be one reason for their less appropriate medical management for AMI [13], [14]. Another possible reason, which has not been tested in extant literature, is that patients with mental comorbidities may be more likely to be treated in hospitals with lower quality care. The delivery of hospital care is highly variable in the US, [15], [16], [17] and socioeconomically or demographically disadvantaged patients such as poor individuals are shown to have reduced access to high-quality hospital care [18], [19]. Mentally-ill patients may face similar disparities. For example, one previous study reported that mentally-ill patients with coronary heart disease were more likely to be referred to lower-quality cardiac surgeons for the receipt of bypass surgery in New York State [20].

The present study analyzed a national cohort of Medicare patients hospitalized for AMI and determined whether those with comorbid mental illness were more likely to present to hospitals with lower quality of AMI care. We further compared differences in outcomes (length of stay, short-term readmissions, and short-term and long-term mortality) between mentally-ill and mentally-intact patients admitted to hospitals of different quality groups. Hospital quality was measured using the “Hospital Compare” quality indicators developed and published on line by the Centers for Medicare and Medicaid Services (CMS) [15], [16], [17].

## Materials and Methods

### Data Source and Sample

The primary source of data was the 2008 Medicare Provider Analysis and Review (MedPAR) file obtained from the CMS. The MedPAR contains information on all hospitalizations for Medicare fee-for-service beneficiaries. Key data elements include patient demographics, 5-digit zip code of patient residence, primary and up to 9 secondary diagnoses recorded by the International Classification of Disease, Ninth Revision, Clinical Modification (ICD-9-CM) diagnostic codes, primary and up to 5 secondary procedures recorded by ICD-9-CM procedure codes, admission and discharge dates, date of death up to two years after discharge, a unique patient identifier, and the hospital identifier that allows for linkage of the MedPAR to external databases.

Our sample included AMI patients (principal ICD-9-CM diagnostic code 410.xx) aged 65 years or older. A small number of patients with missing gender (<1%) were excluded from the sample. The patient sample was further supplemented by several databases including (1) the 2008 Hospital Compare data to obtain hospital performance on AMI process measures (see below); (2) the 2000 US census file to obtain two socioeconomic measures at the zip code of patient residence (household income and high school graduation rate); (3) the 2008 American Hospital Association (AHA) annual survey file that contains variables of hospital characteristics; and (4) the rural urban commuting area (RUCA) file to define rural vs. urban location of the hospital [21].

### “Hospital Compare” Process Measures

Our primary analyses focused on patient admissions to hospitals with differential AMI quality measured by the “Hospital Compare” indicators. We identified all hospitals reporting AMI process performance in 2008 in a database downloaded from the Hospital Compare website (www.hospitalcompare.hhs.gov; n = 4483). Participation in Hospital Compare by hospitals is voluntary, but because of the financial incentives created by CMS for public reporting, almost all hospitals participated and reported adherence rates for the core process measures of several common conditions including AMI [16]. The 5 core measures for AMI include: (1) use of aspirin within 24 hours of admission; (2) use of aspirin at discharge; (3) use of β-blocker within 24 hours of admission; (4) use of β-blocker at discharge; and (5) use of angiotensin converting enzyme (ACE) inhibitor or angiotensin receptor blocker (ARB) for left ventricular systolic dysfunction. For each measure, hospital performance was assessed as the proportion of eligible AMI patients that received the specific therapy. The 2008 reported adherence rate for each measure was calculated from data of all patients admitted to the hospital in 2007.

We excluded all hospitals for which the total number of eligible AMI patients for each of the five process measures was less than 25 (n = 1547) in accordance with similar methodology employed by CMS and other investigators; these hospitals were considered to have too few cases to allow for a statistically reliable assessment of performance [15], [19], [22]. For each remaining hospital, we then created a composite score of process performance calculated as the sum of number of times a hospital performed the appropriate action across all measures (numerator) divided by the number of opportunities the hospital had to provide appropriate care for eligible patients (denominator) [23], [24]. Hospitals were then divided into 3 groups according to rankings of the composite score: high quality hospitals (hospitals with the composite score >90^th^ percentile), low quality hospitals (hospitals with the composite score <10^th^ percentile), and medium-quality hospitals (other hospitals). In sensitivity analyses we used alternative cutoff points to define the high-quality (80^th^, 75^th^, or 67^th^ percentile) and low-quality (20^th^, 25^th^, or 33^rd^ percentile correspondingly) groups.

### Patient Outcomes

Our secondary analyses focused on several patient outcomes included length of stay, all-cause readmissions to an acute care hospital within 30 days after discharge, mortality within 30 days of AMI admission, and mortality within one year of AMI admission.

### Comorbid Mental Illness

The key independent variable for analyses was mental diagnosis, which was defined based on secondary ICD-9-CM codes 290.xx-319.xx, excluding 305.1x for tobacco use (note that all patients in the sample had a principal diagnosis of AMI). Inpatient cases with coexisting mental disorders were further categorized into three mutually exclusive mental subgroups: psychiatric illness only (ICD-9-CM codes 290.xx-302.xx and 306.xx–319.xx), substance-abuse disorder only (ICD-9-CM codes 303.xx–305.xx, excluding 305.1x), and dual diagnosis (both psychiatric and substance-abuse disorders).

### Patient and Hospital Covariates

We identified the following patient covariates that may affect patient admission patterns and outcomes for AMI: age in years, female gender, race/ethnicity categorized as non-Hispanic white, black, and other (Hispanic, Asian/Pacific Islander, Native American); patient zip-code level median household income and high-school graduation rate; individual medical comorbidities defined according the algorithm developed by Elixhauser et al, [25] comorbid tobacco use; and distances from patient residence to the admitting hospital and to the nearest hospital, which were calculated based on straight-line approximations between zip-code centroids of a patient’s residence and the corresponding hospital [26].

Hospital structural characteristics obtained from the AHA annual survey included total number of beds, profit status (for-profit, non-for-profit, or government owned), major teaching hospital (yes/no), rural versus urban location, and nurse staffing level calculated as the number of full-time equivalent nurses divided by 1000 adjusted patient days [27].

### Statistical Analysis

We performed bivariate analyses to describe patient characteristics and rates of admission to low-quality, medium-quality, and high-quality hospitals by mental diagnosis. Chi-square tests were used to determine differences in proportions, and analyses-of-variance were used to determine differences in means. Similar methods were used to compare hospital characteristics by hospital quality group.

We estimated multinomial logit models to test the independent association of mental illness with admission to low-quality or high-quality hospitals. The dependent variable was a categorical variable defining the 3 hospital quality groups, with the medium-quality group being the reference group (i.e., the likelihood of admission to low-quality [or high-quality] hospitals compared to the likelihood of admission to medium-quality hospitals). We estimated separate models to test the overall effect of mental illness and the effect of subgroups of mental illness (psychiatric only, substance abuse only, and dual diagnosis). All models controlled for the same patient demographic, socioeconomic, and diagnostic characteristics, as well as the two distance measures described above.

Lastly, we estimated separate multivariate models to determine differences in outcomes associated with mental illness and the admitting hospital. We estimated a generalized linear regression model for length of stay assuming Poisson distribution, [28] binary logistic regression models for 30-day readmission and for 30-day mortality separately, and a Cox proportional hazard model for one-year mortality. The independent variables in all models were defined according to mental diagnosis and hospital group categories (i.e., the mentally-ill at low-quality hospitals, the mentally-intact at low-quality hospitals; the mentally-ill at medium-quality hospitals, the mentally-intact at medium-quality hospitals; the mentally-ill at high-quality hospitals, and the mentally-intact at high-quality hospitals), using the mentally-intact patients at high-quality hospitals as the comparison group. All models controlled for the same patient demographic, socioeconomic, and diagnostic characteristics, as well as hospital structural characteristics described above. All analyses were performed using SAS (SAS Institute, Cary, NC) version 9.2.

## Results

Our final example included 287,881 Medicare AMI patients admitted to 2,845 acute care nonfederal hospitals in 2008. Compared with patients with no mental illness, mentally-ill patients were 3 years older (81 vs. 78), more likely to be female (59% vs. 48%), and had more medical comorbidities (Table 1). They also lived slightly nearer to a hospital and traveled shorter (12 vs. 14 miles) for admission.

**Table 1:** Characteristics of Medicare AMI patients, by mental illness.

Characteristic	Mental Illness*	No Mental Illness*
	(N = 41044)	(N = 246837)
**Age in years, mean ± SD**	81.0+8.7	78.1+8.4
**Female, %**	59.3	47.8
**Race/Ethnicity, %**		
** White**	88.1	86.9
** Black**	8.0	8.4
** Other**	3.9	4.7
**Median annual household income at zip code of residence (x$1000), mean ± SD**	42.6±15.5	41.9±15.1
**High-school graduation rate at zip code of residence, mean ± SD**	0.8±0.1	0.8±0.1
**Number of medical comorbidities, %**		
** 0**	3.4	3.2
** 1**	13.8	16.2
** 2**	24.9	30.2
** 3**	27.8	29.5
** ≥4**	30.1	20.8
**Tobacco Use, %**	6.5	6.1
**Distance to the admitting hospital in miles, Mean ± SD**	11.9±16.3	14.1±18.1
**Distance to the nearest hospital in miles, Mean ± SD**	5.4±7.5	6.1±8.5

AMI = acute myocardial infarction; SD = standard deviation.

* P<0.01 for comparisons across mental illness groups based on χ^2^ tests or analyses of variance.

Table 2 shows that the average composite quality score was 77.3% for low-quality hospitals, 95.6% for medium-quality hospitals, and 99.9% for high-quality hospitals (p<0.01 for difference). Similar differences were found for individual process scores. Compared to medium-quality or high-quality hospitals, hospitals with low reported quality for AMI tended to be small non-teaching, government-owned, and rural hospitals with lower nurse staffing levels.

**Table 2:** Hospital characteristics by quality ranking group.

Characteristic	Low-quality hospitals, <10^th^ percentile (n = 285)*	Medium-quality hospitals, 10^th^–90^th^ percentile (n = 2275)*	High-quality hospitals, >90^th^ percentile (n = 285)*
**Composite quality score, mean ± SD, %**	77.3±7.7	95.6±3.3	99.9±0.2
** Aspirin at admission**	83.2±11.1	97.0±3.5	99.9±0.4
** Aspirin at discharge**	76.5±14.0	95.2±5.9	99.9±0.3
** ACE-I or ARB for LV dysfunction**	70.1±32.2	91.1±14.1	99.7±1.2
** Beta-blocker at admission**	72.4±13.5	94.0±5.7	99.7±0.6
** Beta-blocker at discharge**	76.6±16.3	96.2±5.0	99.9±0.2
**Number of beds, mean ± SD**	89.8±58.7	246.6±205.6	217.6±181.3
**Profit status, %**			
** For-profit**	20.7	16.9	17.5
** Not-for-profit**	48.4	70.2	71.6
** Government owned**	30.9	12.9	10.9
**Rural urban location, %**			
** Rural**	67.4	25.6	28.4
** Urban**	32.6	74.4	71.6
**Major teaching hospital, %**	0.4	10.8	9.1
**Nurse staffing ratio, mean ± SD**	2.6±1.5	3.0±1.0	3.0±1.5

ACE-I: angiotensin-converting enzyme inhibitor; ARB: angiotensin receptor blocker; LV: left ventricular; SD: standard deviation.

* P<0.01 for all characteristics compared across hospital groups based on χ^2^ tests or analyses of variance.

In bivariate analyses and compared to other AMI patients, AMI patients with comorbid mental illness were more likely to present to low-quality hospitals (2.9% vs. 2.0%) and less likely to present to high-quality hospitals (9.7% vs. 10.3%, Table 3). In multivariate analyses controlling for patient characteristics and distances, the associations remained for admissions to low-quality hospitals (adjusted odds ratio [OR] of mental illness 1.25, 95% confidence interval [CI] 1.17–1.34, p<0.01), but not for admissions to high-quality hospitals (adjusted OR 0.97, 95% CI 0.93–1.01, p = 0.11). We performed sensitivity analyses where different cutoffs were used to categorize hospitals (see Tables S1 and S2); the associations of mental illness with admissions to low- and high-quality hospitals were similar.

**Table 3:** Admission to hospitals with low and high composite quality scores by Medicare acute myocardial infarction patients.

		Low-quality ranking hospitals*			High-quality ranking hospitals*	
	Unadjusted admissionrate, %	Adjusted Odds Ratio (95% CI)**	AdjustedP-Value	Unadjusted admission rate, %	Adjusted Odds Ratio (95% CI)**	Adjusted P-Value
**Mental illness (n = 41044)**	2.9	1.25 (1.17,1.34)	<0.01	9.8	0.97 (0.93,1.01)	0.11
**Psychiatric only (n = 38848)**	2.9	1.25 (1.17,1.34)	<0.01	9.7	0.97 (0.93,1.01)	0.11
**Substance abuse only (n = 1644)**	1.8	0.99 (0.67,1.46)	0.97	9.7	0.92 (0.77,1.11)	0.39
**Dual diagnosis (n = 552)**	3.1	1.89 (1.15,3.11)	0.01	11.1	1.16 (0.87.1.57)	0.32
**No mental illness (n = 246837)**	2.0	1.00	–	10.3	1.00	–

* Defined as hospitals in the bottom (low quality) or top (high quality) 10% rankings of the composite quality score.

** Multivariate multinomial logistic models adjusted for patient age, gender, race, median household income, high school graduation rate, tobacco use, distances to the admitting hospital and to the nearest hospital, and individual medical comorbidities (congestive heart failure, cardiac arrhythmias, valvular disease, pulmonary circulation disorders, peripheral vascular disorders, hypertension, paralysis, other neurological disorders, chronic pulmonary disease, diabetes, hypothyroidism, renal failure, liver disease, peptic ulcer disease excluding bleeding, lymphoma, metastatic cancer, solid tumor without metastasis, rheumatoid arthritis, coagulopathy, obesity, weight loss, fluid and electrolyte disorders, blood loss anemia, and deficiency anemia).

In further analyses of subgroups of mental diagnoses, patients with psychiatric illness only, who made up the majority of mentally-ill patients, showed similar admission patterns, with adjusted OR of 1.25 (95% CI 1.17–1.34, p<0.01) for admissions to low-quality hospitals, and adjusted OR of 0.97 (95% CI 0.93–1.01, p = 0.11) for admissions to high-quality hospitals. Diagnosis of substance abuse only or dual diagnosis did not show significant associations with admissions to either type of hospitals, except that dual diagnosis significantly predicted higher risk of admissions to low-quality hospitals (adjusted OR 1.89, 95% CI 1.15–3.11, p = 0.01); this effect of dual diagnosis, though, was not consistently found in sensitivity analyses (Table S1).

Table 4 shows that the length of stay for AMI was similar across groups defined by admitting hospital quality and patient mental status (e.g., average LOS approximately 6 days). AMI patients admitted to low-quality hospitals had higher 30-day readmission rate than those admitted to medium- or high-quality hospitals (27% vs. 23% vs. 23%). Compared to non-mentally-ill AMI patients admitted to high-quality hospitals, non-mentally-ill patients to low-quality hospitals showed 18% higher risk for readmission within 30 days of discharge (adjusted OR 1.18, 95% CI 1.06–1.30, p<0.01). Hospital quality and mental comorbidity both seemed to affect mortality (short-term or long-term). For example, compared to non-mentally-ill patients admitted to high quality hospitals, mentally-ill patients admitted to low-quality hospitals had 23% increased risk for death in 30 days (unadjusted rate 21.5% vs. 12.8%; adjusted OR 1.23, 95% CI 1.05–1.44, p<0.01) and death in 1 year (unadjusted rate 46.8% vs. 26.6%; adjusted hazard ratio 1.44, 95% CI 1.32–1.58, p<0.01).

**Table 4:** Outcomes of acute myocardial infarction patients admitted to different hospitals.

	Low-quality hospital**		Medium-quality hospital**		High-quality hospital**	
	251689984Mental illness		Mental illness		Mental illness	
	Yes	No	Yes	No	Yes	No
Length of stay, days						
Mean, median (IQR)	5.5, 5 (3–7)	6.1, 5 (3–8)	5.6, 4 (3–7)	6.1, 4 (3–8)	5.4, 4 (3–7)	5.9, 4 (3–7)
Adjusted β–coef. (95% CI)*	0.02 (−0.01, 0.03)	0.01 (−0.05, 0.02)	−0.05 (−0.06, −0.04)	0.02 (0.01,0.03)	−0.06 (−0.08, −0.05)	–
30-day readmission						
Rate, %	26.2	28.6	23.2	23.5	23.3	22.3
Adjusted OR (95% CI)*	1.13 (0.94,1.35)	1.18 (1.06,1.30)	1.09 (1.04,1.14)	1.06 (1.02,1.10)	1.12 (1.01,1.23)	–
30-day mortality						
Rate, %	21.5	20.9	16.9	13.6	15.9	12.8
Adjusted OR (95% CI)*	1.23 (1.05,1.44)	1.27 (1.16,1.39)	1.22 (1.16,1.29)	1.05 (1.00,1.09)	1.21 (1.09,1.34)	–
1-year mortality						
Rate, %	46.8	41.0	36.4	28.4	34.5	26.6
Adjusted HR (95% CI)*	1.44 (1.32.1.58)	1.26 (1.19,1.33)	1.32 (1.28,1.37)	1.06 (1.03,1.09)	1.34 (1.26,1.42)	–

OR = odds ratio; CI = confidence interval; HR = hazard ratio.

Note: The analyses of length of stay and 30-day readmissions excluded patients who died in hospital or were transferred to another acute care hospital after admission. The analyses of 30-day readmissions also excluded readmissions for rehabilitations and were limited to patients admitted before November 30, 2008.

* Multivariate generalized linear (for length of stay), logistic (for readmissions and 30-day mortality) and Cox proportional hazard (for 1-year mortality) models adjusted for patient age, gender, race, median household income, high school graduation rate, tobacco use, individual medical comorbidities (congestive heart failure, cardiac arrhythmias, valvular disease, pulmonary circulation disorders, peripheral vascular disorders, hypertension, paralysis, other neurological disorders, chronic pulmonary disease, diabetes, hypothyroidism, renal failure, liver disease, peptic ulcer disease excluding bleeding, lymphoma, metastatic cancer, solid tumor without metastasis, rheumatoid arthritis, coagulopathy, obesity, weight loss, fluid and electrolyte disorders, blood loss anemia, and deficiency anemia), and hospital characteristics (including number of beds, profit status, rural vs. urban location, teaching status, and nurse staffing ratio).

** Low-quality hospitals were defined as those in the bottom 10% rankings of the composite quality score, medium-quality hospitals in the middle 80%, and high-quality hospitals in the top 10% rankings of the composite quality score.

## Discussion

We found that among Medicare myocardial infarction patients, comorbid mental illness was associated with a 25% increased risk for admission to hospitals with lowest quality of AMI care according to the “Hospital Compare” process measures. In contrast, patients with comorbid mental illness did not seem disadvantaged in access to high-quality hospitals. Hospital quality and patient mental status did not affect length of stay, but did affect other post-AMI outcomes. Generally, lower hospital quality and mental diagnosis predicted higher risks for readmissions within 30 days of discharge, and mortality within 30 days and 1 year of admission.

The public reporting of hospital performance is expected to empower patients to compare and choose hospital services based on quality, and in turn stimulate facility’s internal quality improvement [29]. Due to the emergency nature of AMI, AMI patients may not have much discretion to choose among hospitals, and after the onset of symptoms they usually are sent to the nearest hospital for timely admission and treatment initiation. However, previous evidence suggests that AMI patients may be able to bypass the nearest hospital and choose to be admitted to a preferred although more distant hospital, if their condition is relatively stable and if the preferred institution is not much further away [30], [31]. The preferred hospital could be the one with established care relationship with the patient, the one with more advance technologies such as revascularization facilities, and, for well-informed patients, the one with high reputation or service quality for cardiovascular care. Therefore, even for acute conditions such as AMI, the CMS hospital report card is still expected to function as a quality signal facilitating patient choice. Our findings that the distance from patient residence to the admitting hospital was on average longer than to the nearest hospital (Table 1) confirm that some patients did bypass the nearest hospitals, some of which may be of lowest reported quality.

We expect that patients with comorbid mental illness are less able to make informed choices of a preferred hospital by avoiding the nearest one. First, mentally-ill patients tend to seek care late [32] and to have delayed admission for myocardial infarction [33]. Therefore, their pre-admission condition may be less stable to allow for further traveling to a preferred hospital. Moreover, given their cognitive impairment, possible disrupted social and family support, and possible socioeconomic disadvantages associated with mental illness, these patients would be less likely to have access to and actually use the published data to guide their choice of hospitals. For the same reasons, even when they know a preferred hospital or want to avoid a hospital with inferior quality based on the CMS report, they may be less able to express and assert their preferences (to the emergency service personnel, for example) to be admitted to the preferred hospital.

Our findings in Table 3 reveal that compared to other patients, mentally-ill patients were more likely to present to low-quality hospitals, but were equally likely to present to high-quality hospitals. This would suggest that non-mentally-ill AMI patients, when making informed choices, may avoid hospitals with known poor quality, but may not necessarily go to a best-quality hospital given other possible constraints such as substantially increased travel time or costs of care. Our data in Table 2 suggest that this pattern of choice (i.e., selective avoidance of lowest-performing hospitals) is rational because the largest quality gap exists between low-performing and medium-performing (or high-performing) hospitals, while the highest-performing hospitals did not differentiate themselves so much from the large number of hospitals with “medium” reported quality.

This study further reveals that differential admission patterns have important implications for post-AMI outcomes. Consistent with previous reports, we found that patients at low-quality hospitals had higher risk-adjusted odds of short-term readmission and short-term and long-term mortality than patients at higher-quality hospitals, [15], [16], [17], [19] irrespective of patient mental status. Combined with the findings that mentally-ill patients were more likely to present to lower-quality hospitals, our study suggests that site of care, in addition to mental illness and its associated cognitive, social, and behavioral deficits, plays an important role in determining quality of care and subsequently outcomes. This also suggests that the previously documented disparities in AMI care and outcomes among the mentally ill [7], [10] is in part a system problem, and targeted interventions at particular hospitals (e.g., improve the overall quality of low-performing hospitals) and mentally-ill patients (e.g., improve access to high-quality hospitals) would both address the disparities.

This study has several limitations. First, because our analyses were limited to Medicare fee-for-service patients, conclusions of this study may not be generalized to Medicare HMO patients. Second, the prevalence of mental illness could be under-estimated in the AMI cohort due to potential issues of under-recording or faulty recording of ICD-9 diagnoses in the Medicare claims. However, under-identification of mental illness would bias analyses to no group differences in admission patterns and outcomes, and make our findings conservative estimates of the true associations. Finally, our large database analyses do not capture underlying differences in patient preferences, travel modes, or detailed measures of disease urgency and severity. Therefore, differences in site of care and outcomes may be partly mediated by these unobserved factors.

### Conclusion

In conclusion, this national study of Medicare myocardial infarction patients suggests that mental illness was associated with an increased risk for admission to hospitals with lowest quality of AMI care, although mentally-ill patients were equally likely to present to hospitals with best quality compared to other patients. Lower quality of hospital care and mental illness both predicted worse outcomes including readmissions and mortality. Targeted efforts to improve the quality of medical care for mentally-ill Medicare patients are warranted.

## Supporting Information

Table S1 Admission to hospitals with low composite quality scores by Medicare acute myocardial infarction patients*.
(DOCX)

Table S2 Admission to hospitals with high composite quality scores by Medicare acute myocardial infarction patients*.
(DOCX)
