# Supplementary material for: Are AMI Patients with Comorbid Mental Illness More Likely to be Admitted to Hospitals with Lower Quality of AMI Care?
Source: PLoS One. 2013 Apr 2;8(4):e60258. doi: 10.1371/journal.pone.0060258 (PMC3614995; doi:10.1371/journal.pone.0060258)
Supplement: Table S1 — Admission to hospitals with low composite quality scores by Medicare acute myocardial infarction patients*. (DOCX) [file pone.0060258.s001.docx]

**Table S1.** Admission to hospitals with low composite quality scores by Medicare acute myocardial infarction patients*

|  |  |  | **Low-quality ranking hospitals** |  |  |  |
| --- | --- | --- | --- | --- | --- | --- |
|  | **<20^th^ Percentile** |  | **<25^th^ Percentile** |  | **<33^rd^ Percentile** |  |
|  | **Odds Ratio**  **(95% CI)** | **P** | **Odds Ratio**  **(95% CI)** | **P** | **Odds Ratio**  **(95% CI)** | **P** |
| **Mental illness (n=41044)** | 1.14 (1.10,1.19) | <0.01 | 1.15 (1.11,1.20) | <0.01 | 1.11 (1.08,1.15) | <0.01 |
| **Psychiatric only (n=38848)** | 1.14 (1.10,1.19) | <0.01 | 1.15 (1.11, 1.19) | <0.01 | 1.11 (1.07,1.14) | <0.01 |
| **Substance abuse only (n=1644)** | 1.15 (0.94,1.40) | 0.19 | 1.27 (1.07, 1.51) | 0.01 | 1.20 (1.04,1.39) | 0.01 |
| **Dual diagnosis (n=552)** | 1.26 (0.90,1.76) | 0.19 | 1.27 (0.94, 1.72) | 0.12 | 1.10 (0.84,1.44) | 0.50 |
| **No mental illness (n= 246837)** | 1.00 | ---- | 1.00 | ---- | 1.00 | ---- |

*Multivariate multinomial logistic models adjusted for patient age, gender, race, median household income, high school graduation rate, tobacco use, distances to the admitting hospital and to the nearest hospital, and individual medical comorbidities (congestive heart failure, cardiac arrhythmias, valvular disease, pulmonary circulation disorders, peripheral vascular disorders, hypertension, paralysis, other neurological disorders, chronic pulmonary disease, diabetes, hypothyroidism, renal failure, liver disease, peptic ulcer disease excluding bleeding, lymphoma, metastatic cancer, solid tumor without metastasis, rheumatoid arthritis, coagulopathy, obesity, weight loss, fluid and electrolyte disorders, blood loss anemia, and deficiency anemia). Separate models were estimated for alternatively defined hospital groups.
