# Supplementary material for: Are AMI Patients with Comorbid Mental Illness More Likely to be Admitted to Hospitals with Lower Quality of AMI Care?
Source: PLoS One. 2013 Apr 2;8(4):e60258. doi: 10.1371/journal.pone.0060258 (PMC3614995; doi:10.1371/journal.pone.0060258)
Supplement: Table S2 — Admission to hospitals with high composite quality scores by Medicare acute myocardial infarction patients*. (DOCX) [file pone.0060258.s002.docx]

**Table S2.** Admission to hospitals with high composite quality scores by Medicare acute myocardial infarction patients*

|  |  |  | **High-quality ranking hospitals** |  |  |  |
| --- | --- | --- | --- | --- | --- | --- |
|  | **>80^th^ Percentile** |  | **>75^th^ Percentile** |  | **>67^th^ Percentile** |  |
|  | **Odds Ratio**  **(95% CI)** | **P** | **Odds Ratio**  **(95% CI)** | **P** | **Odds Ratio**  **(95% CI)** | **P** |
| **Mental illness (n=41044)** | 0.95 (0.92,0.98) | <0.01 | 0.97 (0.95,1.00) | <0.01 | 0.97 (0.94,0.99) | <0.01 |
| **Psychiatric only (n=38848)** | 0.94 (0.92,0.97) | <0.01 | 0.97 (0.94,0.99) | 0.01 | 0.96 (0.94,0.99) | <0.01 |
| **Substance abuse only (n=1644)** | 1.01 (0.89,1.15) | 0.87 | 1.07 (0.95,1.21) | 0.25 | 0.99 (0.88,1.13) | 0.85 |
| **Dual diagnosis (n=552)** | 1.10 (0.89,1.37) | 0.37 | 1.22 (1.00,1.49) | 0.05 | 1.18 (0.96,1.44) | 0.12 |
| **No mental illness (n= 246837)** | 1.00 | ---- | 1.00 | ---- | 1.00 | ---- |

*See the footnote of Table S1.
